# Interactions Between Gut Microbiota and Acute Restraint Stress in Peripheral Structures of the Hypothalamic–Pituitary–Adrenal Axis and the Intestine of Male Mice

**DOI:** 10.3389/fimmu.2019.02655

**Published:** 2019-11-19

**Authors:** Karla Vagnerová, Martin Vodička, Petra Hermanová, Peter Ergang, Dagmar Šrůtková, Petra Klusoňová, Kateřina Balounová, Tomáš Hudcovic, Jiří Pácha

**Affiliations:** ^1^Institute of Physiology of the Czech Academy of Sciences, Prague, Czechia; ^2^Institute of Microbiology of the Czech Academy of Sciences, Nový Hrádek, Czechia; ^3^Department of Physiology, Faculty of Science, Charles University, Prague, Czechia

**Keywords:** acute restraint stress, gut microbiota, germ-free, mice, HPA axis, intestine, extra-adrenal glucocorticoid synthesis

## Abstract

The gut microbiota play an important role in shaping brain functions and behavior, including the activity of the hypothalamus-pituitary-adrenocortical (HPA) axis. However, little is known about the effect of the microbiota on the distinct structures (hypothalamus, pituitary, and adrenals) of the HPA axis. In the present study, we analyzed the influence of the microbiota on acute restraint stress (ARS) response in the pituitary, adrenal gland, and intestine, an organ of extra-adrenal glucocorticoid synthesis. Using specific pathogen-free (SPF) and germ-free (GF) male BALB/c mice, we showed that the plasma corticosterone response to ARS was higher in GF than in SPF mice. In the pituitary, stress downregulated the expression of the gene encoding CRH receptor type 1 (*Crhr1*), upregulated the expression of the *Fkbp5* gene regulating glucocorticoid receptor sensitivity and did not affect the expression of the proopiomelanocortin (*Pomc*) and glucocorticoid receptor (*Gr*) genes. In contrast, the microbiota downregulated the expression of pituitary *Pomc* and *Crhr1* but had no effect on *Fkbp5* and *Gr*. In the adrenals, the steroidogenic pathway was strongly stimulated by ARS at the level of the steroidogenic transcriptional regulator *Sf-1*, cholesterol transporter *Star* and *Cyp11a1*, the first enzyme of steroidogenic pathway. In contrast, the effect of the microbiota was significantly detected at the level of genes encoding steroidogenic enzymes but not at the level of *Sf-1* and *Star*. Unlike adrenal *Sf-1*, the expression of the gene *Lrh-1*, which encodes the crucial transcriptional regulator of intestinal steroidogenesis, was modulated by the microbiota and ARS and this effect differed between the ileum and colon. The findings demonstrate that gut microbiota have an impact on the response of the pituitary, adrenals and intestine to ARS and that the interaction between stress and the microbiota during activation of glucocorticoid steroidogenesis differs between organs. The results suggest that downregulated expression of pituitary *Pomc* and *Crhr1* in SPF animals might be an important factor in the exaggerated HPA response of GF mice to stress.

## Introduction

Stressful stimuli induce a cascade of events in the hypothalamic–pituitary–adrenal (HPA) axis, which culminate in the secretion of glucocorticoids from the adrenal gland. The HPA axis is a self-regulatory network, utilizing its end-products, corticosterone in rats and mice and cortisol in humans, to regulate its own activity through a negative feedback mechanism at varying levels of the HPA axis (1). Changes in the HPA axis affect many physiological systems, including the immune system (2), and exposure to stressors modulates the pro-inflammatory cytokines and inflammatory pathways in the brain, endocrine glands, and plasma (3).

Studies performed on germ-free (GF) mice and rats showed that stress modifies not only gut microbiota but also vice versa; gut microbiota alter the stress response and brain neurochemistry (4, 5). GF mice exposed to acute restraint stress exhibited an exaggerated response of the HPA axis with elevated plasma adrenocorticotropic hormone (ACTH) and corticosterone levels, and this discrepancy was normalized after colonization of GF mice with commensal bacteria (6). A similar exaggerated response of the HPA axis was observed in response to acute novel-environment stress in GF mice and rats (7, 8). In contrast, treatment with prebiotics (9) or probiotics (10, 11) attenuated the HPA response to acute restraint or forced swim stress, even if this was not confirmed in all studies (12), probably due to strain-specific effects of the probiotic bacteria.

Taken together, these data strongly demonstrate that gut microbiota play a significant role in the activity of the HPA axis, including the plasma level of glucocorticoids. However, it is unknown what microbiota-induced changes underlie the exaggerated HPA axis activity. The signals originating from microbiota must be transmitted to the brain and/or the peripheral tissues that secrete glucocorticoids. These steroids are secreted primarily from the adrenal cortex, but they can also be generated in peripheral tissues such as the intestine via extra-adrenal glucocorticoid synthesis (13) or via regeneration of biologically active glucocorticoids, corticosterone, or cortisol from their inactive 11-oxo derivatives by enzyme 11β-hydroxysteroid dehydrogenase type 1 (11HSD1) (14). Numerous studies have also shown that neural, immune and endocrine pathways interact with each other at various levels, including the brain and adrenal glands, under normal and stress conditions and that a number of neuropeptides, cytokines, and even bacterial ligands are capable modulating glucocorticoid secretion independently of pituitary ACTH (15–17). Therefore, it is conceivable that gut microbiota might affect steroidogenesis of glucocorticoids. Enterocytes express a wide range of innate immune receptors, cytokines and chemokines (18), and cytokines influence the adrenal steroidogenesis (19), the regeneration of glucocorticoids via 11HSD1 (20) and the brain, including the activity of the HPA axis (21). Similarly, adrenal and intestinal extra-adrenal glucocorticoid synthesis is upregulated by systemic administration of endotoxins (22, 23), and activation of innate immune receptors stimulates steroidogenesis in adrenocortical cells (24).

The physiological response to acute stress related to gut bacteria has only sparsely been studied and focused only on upstream stress regulatory pathways in the brain (6–8). Therefore, in the present study, we aimed to reveal the relationship between gut microbiota and acute stress challenge downstream in the HPA axis, within the pituitary and adrenal gland, and in the intestine, which expresses the machinery of local glucocorticoid synthesis regulated by tumor necrosis factor α (TNFα) (13, 25).

## Materials and Methods

### Animals

Nine-week-old germ-free (GF) and specific pathogen-free (SPF) male BALB/c mice (Institute of Microbiology of the Czech Academy of Sciences, Nový Hrádek, Czechia) were split into four groups: unstressed GF (*n* = 10), stressed GF (*n* = 10), unstressed SPF (*n* = 10), and stressed SPF (*n* = 10) mice. The animals were kept under a 12-h light/dark cycle and were given free access to autoclaved tap water and an irradiated (50 kGy) sterile pellet diet Altromin 1414 (Altromin, Lage, Germany). The GF animals were kept under sterile conditions in Trexler-type isolators since birth and their sterility was assessed every week by microbial cultivation and staining methods. The absence of bacteria, molds, and yeast was confirmed by aerobic and anaerobic cultivation of mouse feces and swabs from the isolators. Germfree status of the mice was further confirmed by the cecal size, weight, and bacterial DNA content when the GF mice were used in the experiments. Breeding of animals in isolators represents very specific environment in terms of handling, exposure to staff, noise level, air pressure etc. In order to ensure equal conditions for all groups during the experiment, the SPF mice were transferred to identical isolator as GF mice 1 month before the beginning of the experiments and were kept under the same conditions as GF mice, i.e., they were fed a sterile diet, drunk autoclaved water, were reared on the sterile bedding and were manipulated by the same staff as the GF mice. As the transfer of mice out of the isolator through a sterilized transfer port via an autoclave jar is a stressful procedure, control mice were transferred into sterile “individually ventilated cages” equipped with a filter system (IVC box; Tecniplast S.p.A., Buguggiate, Italy) 1 week before the end of the experiment. In preliminary experiments, we showed that the transfer of mice from the isolator through the transfer port increased the plasma level of corticosterone from 17.4 ± 4.7 to 103.3 ± 14.3 ng/ml. To minimize the effect of diurnal factors, the mice were stressed between 9:00 and 11:00 and sacrificed between 11:00 and 13:00. The experiments were approved by the Committee for the Protection and Use of Experimental Animals of the Institute of Microbiology of the Czech Academy of Sciences.

### Acute Restraint Stress

The GF and SPF animals were subjected to a 2-h restraint stress in 50-ml conical centrifuge tubes equipped with multiple ventilation holes (26). First, the mice were inserted into the restrainer in the isolator and then during the restraint session, they were transferred out of the isolator through the sterilized transfer port. Immediately after the stress period, the mice were anesthetized with isoflurane vapor, blood was collected by cardiac puncture in K_3_EDTA coated tubes (Sarstedt, Nümbrecht, Germany), centrifuged and the plasma was stored at −80°C before being assayed. Anesthetized mice were decapitated, and the pituitary, adrenal gland, ileum and colon were harvested and snap-frozen in liquid nitrogen for assessment of mRNA expression. Isoflurane was used as an anesthetic because it does not interfere with gene transcriptional responses and leaves the stress response intact (27).

### Sample Preparation and Gene Expression Analysis

Total RNA was extracted from the pituitary, adrenal gland, ileum, and colon using a commercially available kit (Quick-RNA Miniprep Plus, ZYMO Research, Irvine, CA, USA) according to the manufacturer's instructions and quantified by spectrophotometry using a NanoDrop 1000 spectrophotometer (NanoDrop Technologies, Wilmington, DE, USA). First-strand cDNA was prepared from total RNA using random hexamers and a High Capacity cDNA Reverse Transcription Kit (Life Technologies, Carlsbad, CA, USA). Quantitative RT-PCR was carried out using the LightCycler 480 PCR System (Roche Diagnostic GmbH, Mannheim, Germany), 5x Hot Firepol Probe QPCR Mix Plus (ROX) (Solis BioDyne, Tartu, Estonia) and the primers and probes specific for studied transcript (TaqMan Assays, Life Technologies; Generi Biotech, Hradec Králové, Czechia). The following assays were used: pro-opiomelanocortin (*Pomc*, Mm00435874_m1), co-chaperone FK506 binding protein 5 (*Fkbp5*, Mm00487401_m1), corticotropin-releasing hormone (CRH) receptor type 1 (*Crhr1*, Mm00432670_m1), corticotropin-releasing hormone receptor type 2 (*Crhr2*, Mm00438308_m1), glucocorticoid receptor (*Gr*, Mm00433832_m1), melanocortin-2 receptor (*Mc2r*, Mm01262510_m1), steroidogenic acute regulatory protein (*Star*, Mm00441558_m1), lymph node protein 64, a functional homolog of StAR (*Mln64*, Mm00445524_m1), cholesterol side-chain cleavage enzyme (*Cyp11a1*, Mm00490735_m1), 3β-hydroxysteroid dehydrogenase type 1, the major isoform expressed in adrenal gland (*Hsd3b1*, Mm01261921_mH), 3β-hydroxysteroid dehydrogenase type 2, the isoform predominantly expressed in extra-adrenal tissues (*Hsd3b2*, Mm00462685_m1), 11β-hydroxylase (*Cyp11b1*, Mm01204952_m1), steroidogenic factor-1 (*Sf-1*, Mm00446826-m1), liver receptor homolog-1 (*Lrh-1*, Mm00446088), and tumor necrosis factor α (*Tnf*α, Mm00443258_m1). For PCR amplification of 21-hydroxylase (*CYP21a1*) were used the following primers: sense TGGTGCTAAATTCTAACAGA and antisense CTTCCACATGAGAGAGTAATC; probe: ACAGGTCCAAGTCCATCTTTCCAT. To identify the stability of the reference genes, a panel of 12 potential reference genes was compared using geNorm analysis, and the genes *Hprt1* (hypoxanthine-guanine phosphoribosyltransferase 1, Mm01545399-m1) and *Tbp* (TATA-box binding protein, Mm00446973_m1) were identified as the optimal combination to provide reliable normalization in the ileum and colon and *Ppib* (peptidylprolyl isomerase B, cyclophilin B, Mm00478295_m1) and *Sdha* (succinate dehydrogenase subunit A, Mm01352366_m1) in the pituitary and adrenal glands. The expressions of the genes of interest were calculated relative to the geometric mean of the reference genes in each sample. The quantity of the PCR product was determined using the standard curve method with 3-fold dilutions of the mixed cDNA sample.

### Corticosterone Assay

Plasma corticosterone levels were determined by a commercially available Corticosterone rat/mouse ELISA KIT (AR E-8100, LDN GmbH, Nordhorn, Germany). The samples for the assay were determined in a single run to prevent inter-assay variability according to the manufacturer's instructions. The sensitivity of the corticosterone assay was 6.1 ng/ml.

### Statistical Analysis

For statistical comparison, the STATISTICA 9 software package (StatSoft Inc., Tulsa, OK, USA) was used. The data were analyzed by two-way analysis of variance (ANOVA; main factors: microbial status and stress treatment). If there was not a significant interaction effect between both factors, the interaction term was removed from the model and the main effects ANOVA was run. Follow-up comparisons of the means comprising main effects or simple effects of significant interactions were conducted using Tukey's test. The data were expressed as the mean ± SEM, and *p* < 0.05 were considered statistically significant.

## Results

### Effect of Microbiota on Plasma Corticosterone Level in Response to Acute Restraint Stress

**Figure 1:**
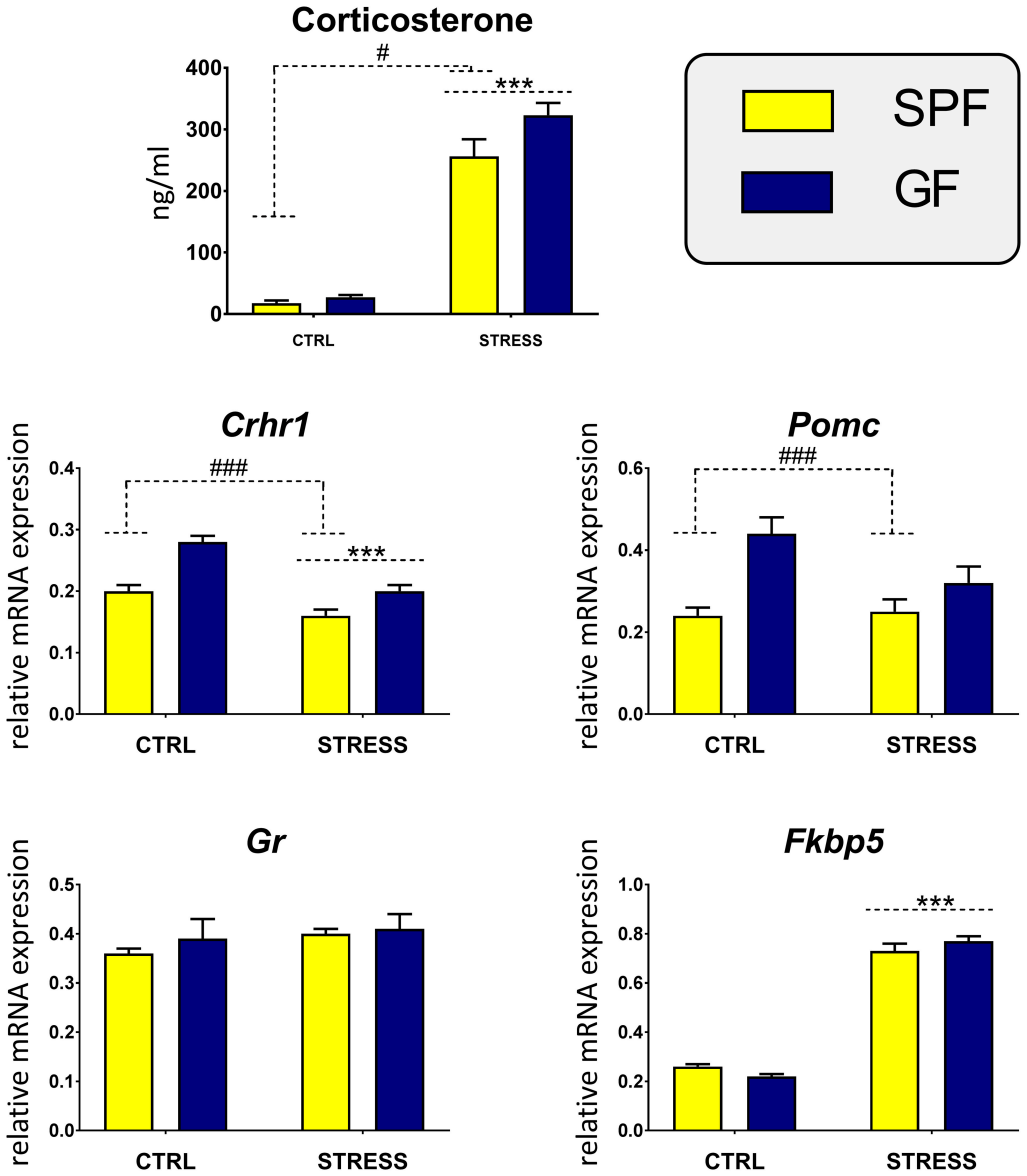
Effect of acute restraint stress on plasma corticosterone and on genes participating in secretion of adrenocorticotropic hormone in the pituitary of specific pathogen-free (SPF) and germ-free (GF) mice. CTRL, unstressed mice; STRESS, mice exposed to restraint stress for 2 h; *Crhr1*, corticotropin-releasing hormone receptor type 1; *Pomc*, pro-opiomelanocortin; *Gr*, glucocorticoid receptor; *Fkbp5*, co-chaperone FK506 binding protein 5. Data are shown as mean ± SEM. A main effect of stress has been identified by placing a dashed horizontal line with an asterisk (^***^p < 0.001) above the bars for the stress-exposed groups, whereas a main effect of microbiota by placing a dashed horizontal line with a hash sign (^###^*p* < 0.001, ^#^*p* < 0.05) above the bars for the SPF groups. No interaction effects between stress and microbiota were observed in any of the analyses.

The two-way ANOVA revealed a significant effect of stress [*F*_1,34_ = 283.45, *p* < 0.001] and microbiota [*F*_1,34_ = 4.82, *p* = 0.035] but no significant interaction effect between the factors. *Post-hoc* analysis demonstrated that both stress and microbiota resulted in increased plasma level of corticosterone (Figure 1).

### Effect of Microbiota on Pituitary Response to Acute Restraint Stress

To establish the impact of microbiota on glucocorticoid and neuropeptide signaling pathways in the pituitary, we examined the expression of the *Crhr1* gene encoding the CRHR1 receptor, whose activation enhances the transcription of *Pomc*, a gene encoding the ACTH precursor (Figure 1). Microbiota had a main effect on both *Crhr1* and *Pomc* expression [*Crhr1*: *F*_1,47_ = 24.42, *p* < 0.001; *Pomc*: *F*_1,47_ = 17.26, *p* < 0.001], but a significant effect of stress was revealed only for the expression of *Crhr1* [*F*_1,47_ = 37.53, *p* < 0.001] but not for *Pomc*. No significant interaction between stress and microbiota was observed in either case. The expression of *Crhr1*, which has been suggested together with the hormones CRH and ACTH to be critical for initiating the stress response, was significantly decreased in stressed mice. Similarly, microbiota downregulated the expression of *Crhr1* and *Pomc*.

To assess the potential differences in the pituitary glucocorticoid feedback between GF and SPF mice, we measured the expression of *Gr* and *Fkbp5*, which encode the glucocorticoid receptor and the co-chaperone participating in the regulation of glucocorticoid receptor sensitivity and in the efficiency of the negative feedback pathway of the HPA axis (28) (Figure 1). Within the pituitary, two-way ANOVA revealed a significant effect of acute stress on the expression of *Fkbp5* [*F*_1,47_ = 556.11, *p* < 0.001], without any significant effect of microbiota or the stress × microbiota interaction. As shown in Figure 1, stress upregulated *Fkbp5* compared with unstressed counterparts. Neither stress nor microbiota modulated the expression of *Gr*.

### Effect of Microbiota and Acute Restraint Stress on Expression of the ACTH Receptor and Steroidogenesis Enzymes in Adrenal Glands

**Figure 2:**
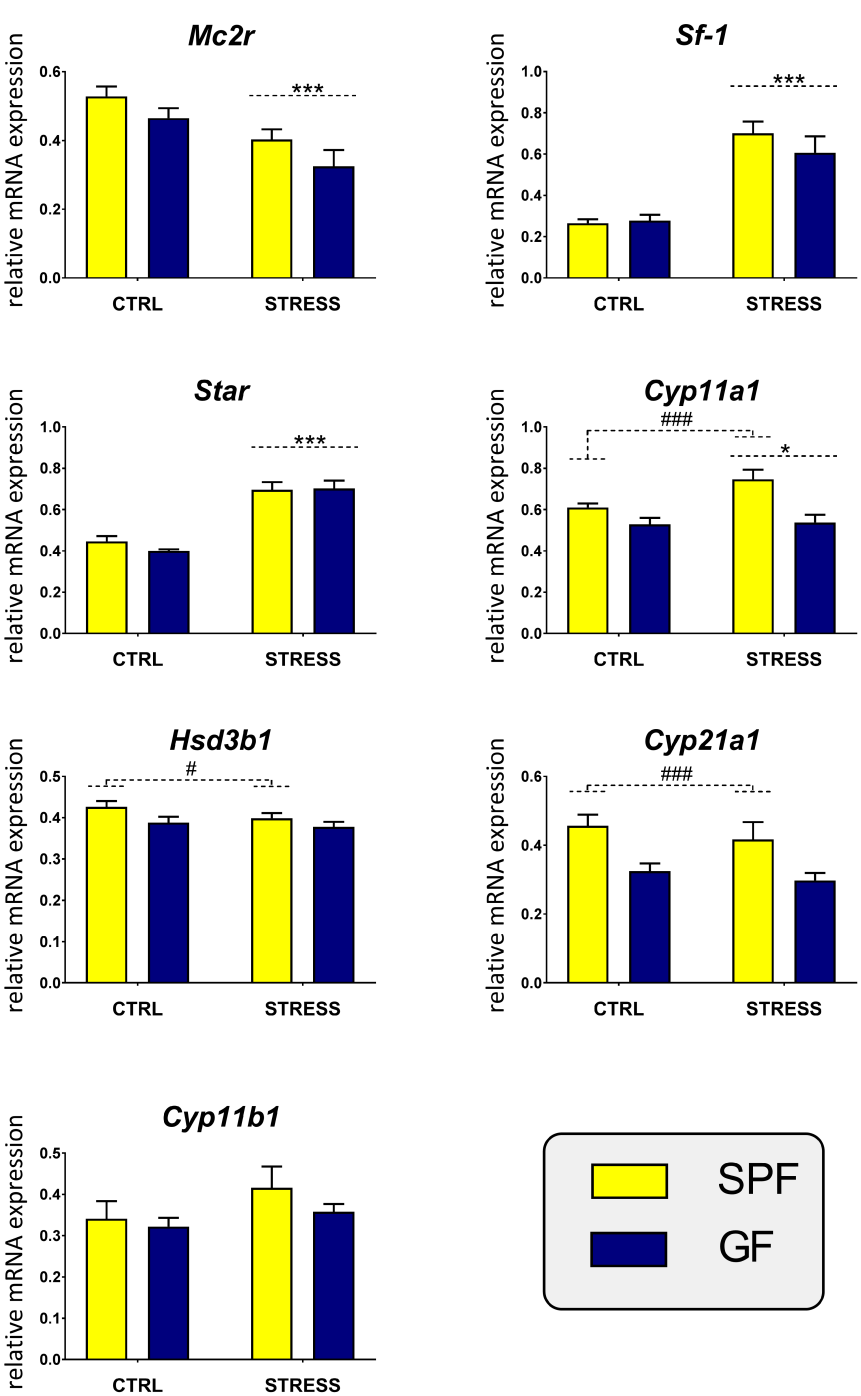
Effect of microbiota and acute restraint stress on the expression of genes participating in adrenal steroidogenesis and its regulation. SPF, specific pathogen-free mice; GF, germ-free mice; CTRL, unstressed mice; STRESS, mice exposed to restraint stress for 2 h; *Mc2r*, melanocortin-2 receptor; *Sf-1*, steroidogenic factor 1; *Star*, steroidogenic acute regulatory protein, *Cyp11a1*, cholesterol side-chain cleavage enzyme, *Hsd3b1*, 3β-hydroxysteroid dehydrogenase type 1; *Cyp21a1*, 21-hydroxylase; *Cyp11b1*, 11β-hydroxylase. All data are expressed as mean ± SEM. A main effect of stress has been identified by placing a dashed horizontal line with an asterisk (^***^p < 0.001, ^*^p < 0.05) above the bars for the stress-exposed groups, whereas a main effect of microbiota by placing a dashed horizontal line with a hash sign (^###^*p* < 0.001, ^#^*p* < 0.05) above the bars for the SPF groups. No interaction effects between stress and microbiota were observed in any of the analyses.

To evaluate the effect of microbiota on the acute stress response in the adrenal glands, the expression of genes participating in adrenal steroidogenesis was quantified, namely, the genes encoding the ACTH receptor (*Mc2r*), a critical transcriptional factor regulating adrenal steroidogenesis (*Sf-1*), a protein that triggers the flow of cholesterol to the steroidogenic machinery (*Star*) and the steroidogenic enzymes (*Cyp11a1, Hsd3b1, Cyp21a1, Cyp11b1*) (29) (Figure 2). A two-way ANOVA of these transcripts did not indicate any statistically significant interaction effect of stress and microbiota. In contrast, the analysis proved the main effect of stress on the expression of genes encoding the first regulatory elements of the steroidogenic pathway, whereas the subsequent elements of this pathway were modulated by microbial status but not by acute stress. Namely, the results showed that *Mc2r* was dependent on stress [*F*_1,34_ = 14.47, *p* < 0.001] but not on microbiota and stress significantly downregulated *Mc2r* expression. Similarly, the expression of adrenal genes, which are known to respond to acute stress, showed a main effect of stress [*Star*: *F*_1,34_ = 79.30, *p* < 0.001; *Sf-1*: *F*_1,34_ = 52.23, *p* < 0.001] accompanied by upregulation of the expression. No significant main effect of stress was found in the expression of genes encoding the enzymes of glucocorticoid synthesis excepting *Cyp11a1* [*F*_1,35_ = 4.21, *p* = 0.048], which was weakly increased by stress. In contrast, microbiota had a main effect on the expression of *Cyp11a1* [*F*_(1, 35_ = 14.96, *p* < 0.001], *Hsd3b1* [*F*_1,35_ = 5.08, *p* = 0.031], and *Cyp21a1* [*F*_1,35_ = 13.83, *p* < 0.001] and *post hoc* tests revealed upregulation of all three genes by gut microbiota. No change due to microbiota was seen in *Cyp11b1*.

### Effect of Microbiota and Acute Restraint Stress on Expression of Genes Encoding Intestinal Biogenesis

**Figure 3:**
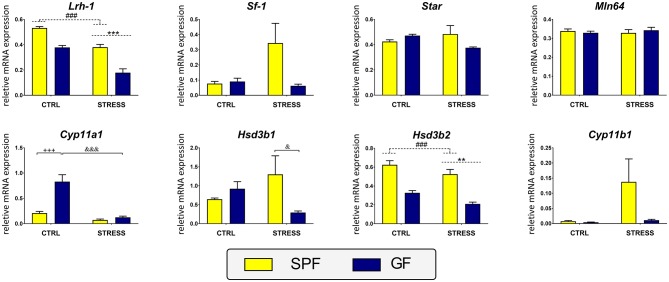
Effect of microbiota and acute restraint stress on the expression of genes participating in colon steroidogenesis and its regulation. SPF, specific pathogen-free mice; GF, germ-free mice; CTRL, unstressed mice; STRESS, mice exposed to restraint stress for 2 h; *Lrh-1*, liver receptor homolog-1; *Sf-1*, steroidogenic factor 1; *Star*, steroidogenic acute regulatory protein; *Mln64*, lymph node protein 64; *Cyp11a1*, cholesterol side-chain cleavage enzyme; *Hsd3b1*, 3β-hydroxysteroid dehydrogenase type 1; *Hsd3b2*, 3β-hydroxysteroid dehydrogenase type 2; *Cyp11b1*, 11β-hydroxylase. All data are expressed as mean ± SEM. Where an interaction effect was observed, the ampersand sign (^&&&^*p* < 0.001, ^&^*p* < 0.05) indicates a significant difference from the stressed GF mice and the plus sign (^+++^*p* < 0.001) indicates a significant difference relative to the unstressed SPF animals. Where no interaction effect was observed, a main effect of stress has been identified by placing a horizontal dashed line with an asterisk (^***^p < 0.001, ^**^p < 0.01) above the bars for the stress-exposed groups, whereas a main effect of microbiota by placing a dashed horizontal line with a hash sign (^###^*p* < 0.001) above the bars for the SPF groups.

As acute inflammatory stress upregulates glucocorticoid synthesis in the intestine (13), we determined the effects of microbiome and stress on the expression of genes encoding selective enzymes and regulatory factors associated with steroidogenesis in GF and SPF mice. Two-way ANOVA revealed that both microbiota and stress modulate the expression of several genes associated with intestinal steroidogenesis. In the colon, the ANOVA indicated a significant interaction between the effect of stress and microbiota on the expression of *Star, Cyp11a1*, and *Hsd3b1* (Table 1). As shown in Figure 3, the *post hoc* analysis revealed a significant stress-dependent decrease of *Cyp11a1* in GF but not SPF mice and the stimulatory effect of germ-free status only in unstressed but not stressed animals. In contrast, germ-free status decreased the response of *Hsd3b1* to stress but did not modulate the expression in unstressed animals. There was no significant effect of a stress × microbiota interaction in other genes participating in intestinal steroidogenesis (Table 1). However, there were significant main effects of stress and microbiota on the expression of *Lrh-1*, encoding a functional homolog of adrenal *SF-1* in the intestine (30) and *Hsd3b2*, the second enzyme of the steroidogenic pathway (Table 1). The presence of microbiota led to the upregulation and stress to the downregulation of *Lrh-1* and *Hsd3b2* (Figure 3). In the case of *Cyp11b1*, only the effect of stress but not microbiota approached significance (Table 1).

**Table 1:** Results of two-way analysis of variance comparing the effects of microbiota and acute restraint stress in the intestine.

	**Colon**				**Ileum**			
	**Df**	**Microbiota**	**Stress**	**Interaction**	**Df**	**Microbiota**	**Stress**	**Interaction**
*Lrh-1*	1, 36	**<0.001** (69.64)	**<0.001** (68.29)	NS	1, 34	0.816 (0.05)	**<0.001** (16.56)	**0.003** (10.10)
*Sf-1*	1, 33	0.095 (2.95)	0.109 (2.71)	NS	1, 28	0.051 (4.15)	0.171 (1.98)	NS
*Star*	1, 32	0.388 (0.77)	0.603 (0.28)	**0.035** (4.84)	1, 33	0.784 (0.07)	0.704 (0.15)	NS
*Mln64*	1, 37	0.864 (0.03)	0.897 (0.02)	NS	1, 33	**0.045** (4.36)	**0.002** (11.49)	**0.039** (4.62)
*Cyp11a1*	1, 35	**<0.001** (20.61)	**<0.001** (32.14)	**<0.001** (14.79)	1, 33	0.161 (2.06)	0.298 (1.12)	NS
*Hsd3b1*	1, 33	0.164 (2.03)	0.960 (0.01)	**0.017** (6.27)	1, 20	0.140 (2.37)	0.182 (1.92)	NS
*Hsd3b2*	1, 37	**<0.001** (71.45)	**0.005** (8.88)	NS	1, 36	**0.003** (9.78)	0.931 (0.01)	NS
*Cyp11b1*	1, 28	0.082 (3.25)	**0.038** (3.00)	NS	1, 34	0.169 (1.97)	0.173 (1.94)	NS
*Tnfα*	1, 37	**0.046** (5.21)	**<0.001** (17.37)	NS	1, 33	**0.009** (7.74)	**0.035** (4.86)	**0.006** (8.71)
*Crhr1*	1, 34	0.517 (0.43)	0.616 (0.25)	NS	1, 25	0.147 (2.23)	0.358 (0.88)	NS
*Crhr2*	1, 36	0.143 (2.24)	**<0.001** (50.62)	NS	1, 32	0.801 (0.06)	0.157 (2.10)	**0.007** (8.37)
*Mc2r*	1, 26	0.108 (2.77)	0.107 (2.79)	**0.035** (4.63)	1, 27	0.095 (2.99)	0.907 (0.01)	NS

*The data represent p-values with bolding indicating a statistically significant main effect or interaction effect; F values are given in parentheses; Df, degrees of freedom; NS, no significant interaction effect between microbiota and stress*.

**Figure 4:**
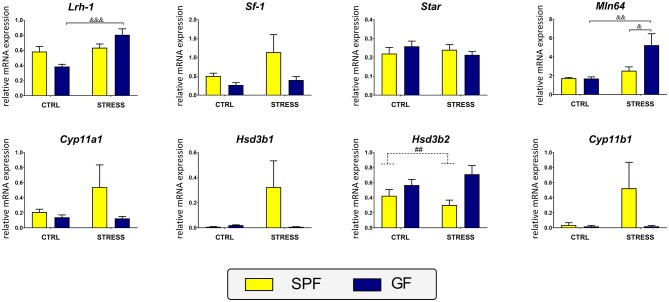
Effect of microbiota and acute restraint stress on expression of genes participating in ileal steroidogenesis and its regulation. For further details, see the description of Figure 3. All data are expressed as mean ± SEM. Where an interaction effect was observed, the ampersand sign (^&&&^*p* < 0.001, ^&&^*p* < 0.01, ^&^*p* < 0.05) indicates a significant difference from the stressed GF mice. Where no interaction effect was observed, a main effect of microbiome has been identified by placing a dashed horizontal line with a hash sign (^##^*p* < 0.01) above the bars for the SPF groups. No main effects of stress were found in any of the analyses.

Within the ileum, the main effect of microbiota approached significance only on the expression of *Hsd3b2* and the effect of stress × microbiota interaction on *Lrh-1* and *Mln64* (Table 1). As shown in Figure 4, microbiota downregulated the expression of *Hsd3b2* and stress upregulated *Lrh-1* and *Mln64*, but here the effect depended on the absence of microbiota.

### Effect of Microbiota and Acute Restraint Stress on Expression of TNFα and Melanocortin and CRH Signaling in the Intestine

**Figure 5:**
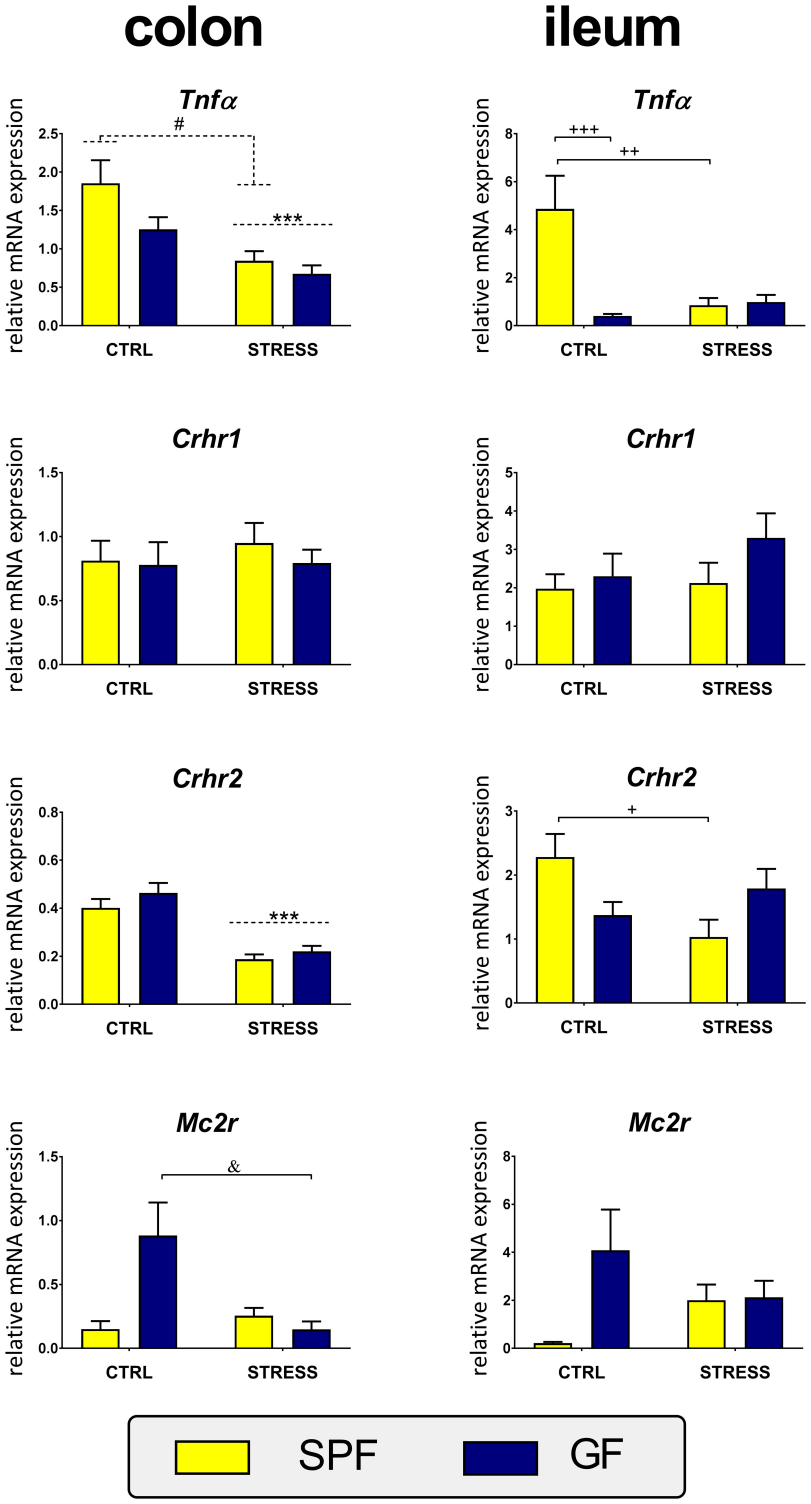
Effect of stress on expression of the genes encoding tumor necrosis factor α and receptors for adrenocorticotropic and corticotropin-releasing hormones in the ileum and colon of specific pathogen-free (SPF) and germ-free (GF) mice. CTRL, unstressed mice; STRESS, mice exposed to restraint stress for 2 h; *Tnf*α, tumor necrosis factor α; *Crhr1*, corticotropin-releasing hormone receptor type 1; *Crhr2*, corticotropin-releasing hormone receptor type 2; *Mc2r*, melanocortin-2 receptor. All data are expressed as mean ± SEM. Where an interaction effect was observed, the ampersand sign (^&^*p* < 0.05) indicates a significant difference from the stressed GF mice and the plus sign (^+++^*p* < 0.001, ^++^*p* < 0.01, ^+^*p* < 0.05) indicates a significant difference relative to the unstressed SPF animals. Where no interaction effect was observed, a main effect of stress has been identified by placing a dashed horizontal line with an asterisk (^***^p < 0.001) above the bars for the stress-exposed groups, whereas a main effect of microbiota by placing a dashed horizontal line with a hash sign (^#^*p* < 0.05) above the bars for the SPF groups.

Both stress and microbiota affected the expression of *Tnf*α in the colon and ileum. Whereas, the interaction between both factors was not significant in the colon, the two-way ANOVA proved a robust interaction between stress and microbiota in the ileum (Table 1). As shown in Figure 5, stress significantly downregulated and microbiota upregulated the expression of *Tnf*α in the colon. However, in the ileum, stress downregulated *Tnf*α only in SPF but not GF mice, where the expression of the cytokine was very low.

To determine whether microbiota modulate peripheral CRH and melanocortin signaling in acute stress, the expression of the receptors *Crhr1, Crhr2*, and *Mc2r* were compared between stressed and unstressed SPF and GF mice. A significant effect of stress was found on *Crhr2* in the colon and a stress × microbiota interaction on *Crhr2* in the ileum and *Mc2r* in the colon. No effects of stress and microbiota were found on *Crhr1* expression either in the colon or ileum (Table 1). *Post-hoc* tests revealed that stress significantly decreased the expression of *Crhr2* in the colon but in the ileum this effect was observed only in SPF mice (Figure 5). Expression of *Mc2r* showed a similar pattern in both intestinal segments, with a significant downregulation of *Mc2r* expression by stress only in the colon of GF mice (Figure 5).

## Discussion

There is growing evidence that microbiota regulate the responsiveness of the HPA axis to stress. Similar to Sudo et al. (6) and Clarke et al. (7), we showed an exaggerated response of the HPA axis to acute restraint stress in GF mice, but our study extends this finding by demonstrating that the microbiota have a profound modulatory effect not only on brain neurochemistry (6, 8, 31) but also on the pituitary and adrenal glands and extra-adrenal tissues.

Contrary to our expectations, we did not observe higher expression of *Pomc* in the pituitary after acute restraint stress, despite our previous report demonstrating upregulation of pituitary *Pomc* after chronic psychosocial stress (32) and the findings of Aguilera et al. showing increased expression of this gene after 14 days of repeated immobilization (33). This discrepancy may reflect either the different timelines of the experiments or different stressors used. In acute stress, the absence of increased pituitary *Pomc* expression after 45 min of restraint stress was observed in domestic chickens (34), whereas 15 min of restraint upregulated *Pomc* levels in the rat pituitary (35), but after 2 h of restraint, this level was already at the control value (36). The decreased *Crhr1* expression during acute stress is in line with previous findings in rat (37). Nevertheless, the expression of pituitary *Pomc* and *Crhr1* were upregulated in GF animals without any significant effect of microbiota on the expression of *Gr* and *Fkbp5*. This finding differs from our previous results (32), which showed an absence of any effect of microbiota on the expression of *Pomc* and *Crhr1* in the pituitary. This discrepancy seems to reflect differences in the treatment of control groups in both experiments. In our previous experiment, the GF and SPF mice were kept in groups of 4–5 per cage and were transferred from the isolator through a sterilized transfer port, where the animals had to spend some time in the transfer jar, whereas in the current experiment, the mice were kept in sterile IVC boxes and thus were not exposed to acute transfer and handling stress, which increased the instantaneous plasma level of corticosterone (see Materials and Methods). Regarding the effect of stress, the upregulation of pituitary *Fkbp5* and downregulation of *Crhr1* were also described in other studies (32, 38), and this downregulation was connected with the action of microRNA (36). To achieve homeostasis, glucocorticoids suppress the HPA axis through feedback inhibition of hypothalamic CRH and pituitary POMC synthesis and secretion (39). Therefore, the appropriate regulation of adrenal glucocorticoid synthesis is dependent not only on the adrenal responsiveness to ACTH but also on the synthesis and secretion of CRH in the hypothalamus and the degree of glucocorticoid-mediated feedback inhibition of the HPA axis. The absence of any effect of microbiota on *Gr* and *Fkbp5* expression indicates that the efficiency of the negative feedback loop via pituitary glucocorticoid receptors is not modulated by the microbiota. The *Fkbp5* gene encodes a protein that regulates the glucocorticoid-mediated negative feedback loop through a decrease of the corticosterone affinity to glucocorticoid receptors and the trafficking of the receptor ligand complex to the nucleus (28). In contrast to the absence of any effect of microbiota on the glucocorticoid negative feedback loop, the downregulation of pituitary *Pomc* and *Crhr1* expression by microbiota suggests the possibility that the higher expression of *Pomc* and *Crhr1* in GF mice might contribute to the exaggerated HPA response to stress in these animals.

The ACTH-dependent regulation of glucocorticoid production requires the precisely coordinated transcription of a variety of genes involved in numerous aspects of steroidogenesis within the adrenal cortex where the nuclear receptor SF-1 represents the critical mediator, which transcriptionally regulates a variety of steroid biosynthetic enzymes (29). However, although the GF mice showed higher HPA axis reactivity to stress than SPF animals, the genes of the ACTH signaling pathway in the adrenal gland were independent of microbiota, particularly *Mc2r, Sf-1*, and *Star*, a gene whose transcription is rapidly stimulated by ACTH. Despite the effect of microbiota on the expression of genes encoding enzymes of adrenal steroidogenesis, acute restraint stress strongly upregulated *Sf-1* and *Star* but had no significant effect on the expression of steroidogenic enzyme genes with the exception of a weak effect of stress on *Cyp11a1*. Similar resistance of steroidogenic genes to acute restraint stress was shown recently by others (34, 40). These findings are in accordance with rapid stimulation of *Star* transcription during acute regulation of steroidogenesis and with less obvious effect on *Cyp11a1*, whose increased expression is associated predominantly with chronic maintenance of steroidogenesis (29).

The microbiota impact the systemic glucocorticoid response to stress (6–8), but whether the microbiota are involved in stressor-induced extra-adrenal glucocorticoid synthesis is not clear. It has been previously shown that acute inflammation stress increases local secretion of corticosterone from the intestine due to upregulation of *Cyp11a1* and *Cyp11b1* (13). As the design of our study allowed us to evaluate steroidogenesis not only in the adrenal gland but also in other tissues, we further studied whether acute restraint stress and the microbiota modulate intestinal glucocorticoid steroidogenesis. First, we provided evidence in favor of the effect of our stress paradigm in the intestine. Microbiota were shown to upregulate and chronic stress to downregulate *TNF*α mRNA and protein secretion (32). Second, the intestinal CRH system, a well-established regulatory system in the gastrointestinal tract, was shown to respond to various stressors (41). Consistent with this, microbiota upregulated intestinal *Tnf*α expression, and acute restraint stress downregulated *Crhr2* and *Tnf*α. Detailed analysis of steroidogenic genes in the colon showed a profound effect of stress and microbiota on the expression of several genes, particularly *Lrh-1*, whose gene product is a functional homolog of the transcription factor SF-1 and plays a crucial role in the regulation of intestinal steroidogenesis (42). Surprisingly, despite the upregulation of *Sf-1* transcript in the adrenals of stressed animals, we found downregulation of colonic *Lrh-1* by stress in both GF and SPF animals, and this downregulation was not followed by a corresponding decrease in *Star* expression. Stress-induced downregulation was identified only in the case of *Cyp11a1* in GF animals, and the transcripts of all other genes of glucocorticoid synthesis were not significantly downregulated by stress. In contrast to the colon, stress upregulated the expression of *Lrh-1* in the ileum but only in GF mice and the same pattern was observed in the case of *Mln64*, a gene encoding protein that has been implicated in cholesterol transport and steroidogenesis (43).These data are not in line with previous studies, which have shown that acute inflammatory stress upregulated the expression of *Lrh-1, Cyp11a1*, and *Cyp11b1* in the intestine (13, 42). The different responses of steroidogenic genes to acute inflammatory and restraint stress may be because the gene encoding TNFα, a master regulator of intestinal glucocorticoid synthesis during inflammation (25), was either downregulated or unchanged after restraint stress. Final proof regarding, whether acute restraint stress has a similar effect on intestinal synthesis of glucocorticoids as acute inflammatory stress will require further experiments. Nevertheless, the data indicate that acute restraint stress might influence intestinal steroidogenesis and that this effect depends on microbiota. First, the stimulatory effect of stress on the expression of ileal *Lrh-1* and *Mln64* was observed only in GF mice. Second, the expression of *Sf-1*, which activates the promoter of intestinal *Cyp11a1* and *Cyp11b1* similar to *Lrh-1* (30), showed a trend toward a significant increase by a stressor only in SPF animals. Third, the interaction between stress and microbiota determined the expression of *Cyp11a1*.

While our experiments show the impact of gut microbiota on the response of the pituitary, adrenal and intestine to stress, there are several limitations to these data. The current study used only males and thus the impact of sexual dimorphism of HPA axis cannot be excluded. First, compared to males, female mice and rats show a more robust HPA axis response, as a result of circulating estradiol, which elevates stress hormones levels during non-threatening situations and during stress (44). This sexual dimorphism reflects not only differences in the central components of the HPA axis but also in the adrenal responsiveness to ACTH (45–47). However, the mechanisms surrounding the stronger adrenal phenotype of females are not well understood (48–51). Second, recent data indicate that microbiome leads to alterations of sex-dimorphic gene expression (52) but no interaction between stress, sex, and GF status was observed in the release of corticosterone following a novel-environment stressor (7). Therefore, more studies will be necessary to assess whether the effect of stress and microbiota on activation of glucocorticoid steroidogenesis is a sex-specific process.

In conclusion, the findings reported here demonstrate that the microbiota have a significant impact on the response of the peripheral components of the HPA axis and extra-adrenal glucocorticoid steroidogenic pathway to acute restraint stress. In particular, we found that a lower expression of *Pomc* and *Crhr1* in the pituitary of SPF mice could partially explain the exaggerated HPA axis reactivity in GF animals. In contrast, the weak effect of microbiota on the expression of genes of the adrenal glucocorticoid synthetic pathway indicates that the increased reactivity of the HPA axis in GF mice is not related to changes in the expression of adrenal steroidogenic enzymes. Finally, our study revealed that the response of the intestinal extra-adrenal glucocorticoid pathway to acute stressors depends on the microbiota. Although the precise mechanisms by which microbiota mediate these changes have yet to be elucidated, our findings show that the acute stress response is shaped by microbiota not only in the components of the HPA axis but also in peripheral organs and that the activation of intestinal steroidogenesis is controlled differently from that in the adrenals.

## Data Availability Statement

All datasets generated for this study are included in the article/supplementary material.

## Ethics Statement

The experiments were approved by the Committee for the Protection and Use of Experimental Animals of the Institute of Microbiology of the Czech Academy of Sciences.

## Author Contributions

JP, MV, and TH conceived the study, designed the experiments, and led the project. KV, PH, PE, PK, and KB performed the experiments and analyses. DŠ prepared the germ-free animals. JP, KV, and MV wrote the manuscript. All of the authors read and approved the final manuscript.

### Conflict of Interest

The authors declare that the research was conducted in the absence of any commercial or financial relationships that could be construed as a potential conflict of interest. The handling editor declared a past co-authorship with the authors PH, DŠ, and TH.
